# Eosinophilic Asthma: Pathophysiology and Therapeutic Horizons

**DOI:** 10.3390/cells13050384

**Published:** 2024-02-23

**Authors:** Musaddique Hussain, Gang Liu

**Affiliations:** Division of Pulmonary, Allergy, and Critical Care Medicine, Department of Medicine, University of Alabama at Birmingham, Birmingham, AL 35294, USA

**Keywords:** asthma, eosinophil, eosinophilic asthma, biomarkers, inflammation, interleukins

## Abstract

Asthma is a prevalent chronic non-communicable disease, affecting approximately 300 million people worldwide. It is characterized by significant airway inflammation, hyperresponsiveness, obstruction, and remodeling. Eosinophilic asthma, a subtype of asthma, involves the accumulation of eosinophils in the airways. These eosinophils release mediators and cytokines, contributing to severe airway inflammation and tissue damage. Emerging evidence suggests that targeting eosinophils could reduce airway remodeling and slow the progression of asthma. To achieve this, it is essential to understand the immunopathology of asthma, identify specific eosinophil-associated biomarkers, and categorize patients more accurately based on the clinical characteristics (phenotypes) and underlying pathobiological mechanisms (endotypes). This review delves into the role of eosinophils in exacerbating severe asthma, exploring various phenotypes and endotypes, as well as biomarkers. It also examines the current and emerging biological agents that target eosinophils in eosinophilic asthma. By focusing on these aspects, both researchers and clinicians can advance the development of targeted therapies to combat eosinophilic pathology in severe asthma.

## 1. Introduction

Asthma, a chronic respiratory disease, is characterized by symptoms like wheezing, chest tightness, coughing, and shortness of breath, resulting from factors including airway inflammation, bronchoconstriction, hyperresponsiveness, and airway remodeling [1]. This remodeling involves hypertrophy and hyperplasia of the airway smooth muscle cells, leading to reduced lung function and recurrent exacerbations [2]. The pathophysiology of asthma encompasses a multifaceted interplay of molecular and cellular components, ranging from cytoskeletal proteins to inflammatory mediators [3,4,5,6]. Severe asthma, a complex subtype, presents significant treatment challenges despite rigorous therapies like high-dose glucocorticoids, beta-agonists, and muscarinic antagonists [7], affecting about 334 million individuals globally, with 5–10% cases being severe and not responding well to the standard treatments [8].

Eosinophilic asthma, a subtype of asthma characterized by heightened levels of eosinophils in the airways, peripheral blood, and sputum, involves a complex interplay of factors that contribute to its development and progression. Although a standard definition of eosinophilic asthma remains elusive, clinical trials have utilized peripheral blood eosinophil counts of ≥150 cells/µL, ≥300 cells/µL, or ≥400 cells/µ, as well as sputum eosinophil levels exceeding 2 to 3%, to describe eosinophilic asthma and can readily be identified in a primary care setting [7,9,10].

The primary causes of eosinophilic asthma include exposure to allergens such as pollen, dust mites, and pet dander; genetic predisposition; psychological stressors; obesity; and environmental triggers like pollutants and respiratory infections. These environmental and physiological factors trigger an immune response, leading to chronic inflammation in the airways. Chronic inflammation can induce structural changes in the airways, resulting in airway remodeling characterized by thickening of the smooth muscle and increased mucus production. Furthermore, bronchial hyperresponsiveness, a heightened sensitivity of the airways to stimuli, contributes to exaggerated bronchoconstriction and airflow limitation. Impaired lung function, marked by reduced airflow rates and compromised gas exchange, is another consequence of persistent inflammation. 

Normally, eosinophils undergo apoptosis and are cleared by macrophages without causing inflammation. However, disruptions in this apoptotic process can prolong the presence of eosinophils in the airways, exacerbating the manifestations of eosinophilic asthma [11,12]. While the exact prevalence of eosinophilic asthma is uncertain, it is estimated to represent about 50% of all severe asthma cases [13].

This review aims to provide an in-depth exploration of eosinophilic asthma, examining its immunopathology, phenotypes, endotypes, and biomarkers and both the current and emerging therapeutic strategies.

## 2. Immunopathology: From Eosinophil Development to Eosinophilic Asthma

Eosinophils, as specialized granulocytes, play a critical role in driving various inflammatory responses. The development of eosinophils begins with the differentiation of progenitor and hematopoietic stem cells into eosinophil/mast cell progenitors (EoMCPs) within the bone marrow. These EoMCPs then give rise to eosinophil progenitors (EoPs), which mature into eosinophils. The development and maturation of eosinophils from CD34^+^ hematopoietic precursors in the bone marrow are influenced by cytokines such as IL-3, IL-5, and granulocyte-macrophage colony-stimulating factor (GM-CSF) [14]. Among these, IL-5 plays a crucial role in initiating the development, maturation, and survival of eosinophils in the peripheral tissues, although it is not absolutely essential to eosinophil development [15]. There is redundancy in the regulatory mechanisms of IL-5 activity, as it can be produced by various cell types.

Mature eosinophils possess complex surface receptor structures that interact with chemokines, activating factors and inhibitory factors. Chemokines such as eotaxin-1, -2, and -3; RANTES; and PGD2 attract eosinophils to inflammation sites via the receptors CCR3, CCR1, and DP2/CRTh2. The activating signals include IL-5, IL-3, and GM-CSF, which bind to their corresponding receptors (IL-5R, IL-3R, and GM-CSFR). The inhibitory signals comprise Sialyl-Lewis X (CD15s) via Siglec-8 and TGFβ, which interacts with TGFβR. 

During an inflammatory response, both mature eosinophils and their immature progenitors can exit the bone marrow and infiltrate distant tissues within 8–24 h, typically residing in the tissues for about 3–8 days [16]. The adhesion of the eosinophils to the endothelial cells is critical for their entry into peripheral tissues [17]. Molecules such as P-Selectin (CD162, CD62P), L-selectin (CD62L), and a family of integrins (VLA-4, CR3 and 4, LFA1) play crucial roles in eosinophil rolling, stable adhesion, and transmigration. IL-4 and IL-13 primarily influence endothelial cell adhesion, while IL-5 primes the eosinophils for adhesion [14]. Chemokines, including CCL7 (MCP3), CCL5 (RANTES), CCL13 (MCP-4), CCL11 (eotaxin 1), CCL15, CCL26, and CCL24, facilitate the recruitment of eosinophils from the blood into the organs, such as the lungs, by binding to the chemokine receptors CCR3 or CRTH2 on the eosinophils, a process further synergized by IL-5 [18].

The eosinophil count in asthma patients distinguishes between eosinophilic and non-eosinophilic asthma. Eosinophilic asthma is further categorized as atopic eosinophilic asthma and nonatopic eosinophilic asthma, indicating whether it arises from specific allergic atopic reactivity or from non-specific, non-allergic, nonatopic mechanisms. Atopic eosinophilic asthma is typically Th2-high, while nonatopic eosinophilic asthma primarily involves innate lymphoid cell type 2 (ILC-2) [19]. Both lead to similar immunological outcomes, with eosinophils being central to their functional overlap.

**Figure 1:**
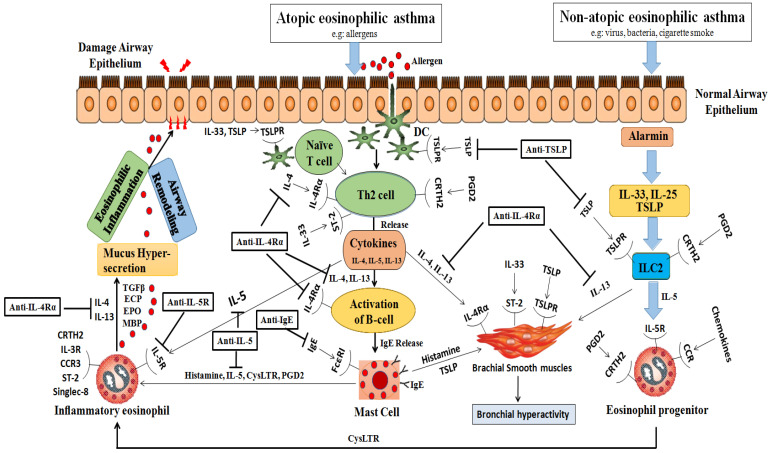
Immunopathology of eosinophilic asthma and targets of biological. This figure illustrates the immunological processes involved in eosinophilic asthma, highlighting the differences between the atopic and nonatopic variants: **Atopic Eosinophilic Asthma:** Initial step: allergens penetrate the airway epithelium after exposure; dendritic cells (DCs): capture these allergens and facilitate the differentiation of Th2 cells; Th2 cell differentiation: leads to the release of multiple cytokines, notably IL-4; IL-4’srole: stimulates B cell activation, leading to IgE release; mast cells: respond by initiating degranulation and releasing histamine, IL-5, cysteinyl leukotrienes (CysLTRs), and prostaglandin D2 (PGD2); eosinophil degranulation: triggered by mast cell factors and further enhanced by IL-4 and IL-13 from the Th2 cells. **Nonatopic Eosinophilic Asthma:** Alarmins: IL-25, IL-33, and thymic stromal lymphopoietin (TSLP) interact with the type 2 innate lymphoid cells (ILC2s) via the CRTH2 receptor; significance of IL-5: this interaction is crucial, particularly in the presence of IL-5 cytokines, leading to the activation or degranulation of eosinophils. Common Outcomes for Both Types: Mucus hypersecretion: augmented due to the combined effects; airway remodeling and eosinophilic inflammation: result of the synergistic action of the described processes; detrimental effects on the airway epithelium: caused by the released interleukins (IL-4, IL-13), eosinophil cationic protein (ECP), eosinophil peroxidase (EPO), and major basic protein (MBP). **Abbreviations:** EOS: eosinophil; DC: dendritic cell; TSLP: thymic stromal lymphopoietin; CysLTR: cysteinyll leukotriene; PGD2: prostaglandin D2; ILC2: type 2 innate lymphoid lcell; ECP: eosinophil cationic protein; EPO: eosinophil peroxidase; MBP: major basic protein; CRTH2: chemoattractant receptor-homologous molecule expressed on Th2 cells; TSLPR: TSLP receptor.

In nonatopic eosinophilic asthmatics, interactions between environmental pollutants, glycolipids, and microbes with the airway epithelium trigger the release of alarmins from activated epithelial cells. These epithelial-derived alarmins stimulate the release of IL-25, IL-33, and TSLP [20]. Eicosanoids, such as PGD2 and cysteinyl leukotrienes C4 and D4, play a crucial role in activating type 2 innate lymphoid cells (ILC2s). Unlike T cells, ILC2s lack T cell receptors (TCRs) but possess receptors responsive to alarmins and leukotrienes. Figure 1 visually represents how eosinophils and immune cells contribute to the development of atopic and nonatopic eosinophilic asthma.

Both Th2 cells (in atopic eosinophilic asthmatics) and ILC2 cells (in nonatopic eosinophilic asthmatics) lead to robust downstream production of type 2 cytokines, particularly IL-4, IL-5, and IL-13. These cytokines are associated with airway eosinophilia, tissue damage, mucous hypersecretion, chronic airway inflammation, and asthma exacerbations. In atopic eosinophilic asthma, IL-4 plays a pivotal role in differentiating naive CD4+ T cells into Th2 cells, promoting the secretion of IL-5 and IL-13, the switching of allergen-specific B cells, and the production of IgE antibodies. IgE interacts with a high-affinity receptor (FcεRI) on the mast cells, leading to mast cell degranulation/activation and the release of mediators such as PGD2, tryptase, and protease, which are associated with increased vascular permeability and vasodilation. Additionally, IL-5 triggers a cascade of intracellular signaling events that are associated with eosinophil recruitment, maturation, activation, and survival, as well as inducing eosinophilic inflammation through the release of toxic granules and cysteinyl leukotrienes [21]. The key signaling molecules in this pathway include PI3K [22]; JAK2; STAT1, 3, and 5 [23]; NF-κB; and MAPK [24]. IL-13 exhibits a versatile role in asthma pathogenesis, including inducing iNOS (inducible nitric oxide synthase) expression, FENO production, mucous hypersecretion, and bronchial hyperactivity by stimulating bronchial smooth muscle cell contraction. In atopic eosinophilic asthma, the cytokines secreted from the mast cells and Th2 cells, particularly IL-4 and IL-13, which share the IL-4Rα receptor, synergistically promote eosinophil recruitment by increasing the expression of adhesion molecules on the endothelial cells and inducing the production of chemokines (eotaxins) by the epithelial cells. The cross-talk between the mast cells and bronchial smooth muscle cells, involving TSLP, histamine, and IL-33, contributes to bronchial hyperactivity.

Eosinophilic extracellular traps (EETs), comprising mitochondrial DNA and eosinophil granule proteins, contribute to allergic disease and asthma pathogenesis. They are released by a process known as extracellular trap cell death (ETosis). ETosis involves NADPH-oxidase-assisted active eosinophil death, releasing eosinophil granules and fibrous nuclear contents. Higher numbers of EET-producing eosinophils correlate with asthma severity, potentially driving Th2-type inflammation via ILC2 stimulation [25]. Additionally, in eosinophilic asthma, the presence of Charcot–Leyden crystals, indicative of eosinophilic inflammation and activity, is often associated with ETosis [26]. These crystals commonly detected in sputum and airway mucus cause bronchial hyperreactivity.

Eosinophils contain a variety of tissue-damaging cationic eosinophil granule proteins, such as eosinophil cationic protein (ECP [RNASE3]), major basic protein (MBP [MBP1 and PRG2]), eosinophil-derived neurotoxin (EDN [RNASE2]), and eosinophil peroxidase (EPX). These proteins can cause inflammation, tissue damage, and mast cell degranulation, leading to the pathogenesis of various airway diseases. Additionally, the TGFβ released from the eosinophils promotes fibroblast proliferation and extracellular matrix production, contributing to airway remodeling [27]. The lipid mediators, like eicosanoids, released from the eosinophils contribute to asthma pathogenesis by releasing the 15(s)-hydroxyeicosatetraenoic acid (15S-HETE) metabolite from the 15-lipoxygenase pathway, leading to airway eosinophilia, increased airway mucus production, and subepithelial remodeling [28].

Eosinophils play a pivotal role in augmenting the T2/Th2 inflammatory pathway by directly or indirectly releasing various cytokines (IL-4, IL-5, IL-13, and IL-25) and chemokines (CCL5/RANTES, CCL11/eotaxin, and CCL3) [29]. In both atopic and nonatopic eosinophilic asthma, various mediators, including tissue-damaging proteins, lipid mediators, chemokines, and cytokines released by the eosinophils, collectively contribute to airway remodeling, airway hyperreactivity, and increased mucus production.

## 3. Clinical Phenotypes of Eosinophilic Asthma

Eosinophilic asthma can be clinically manifested in two main forms: atopic and nonatopic. Within these categories, eosinophilic asthmatics are further divided into (a) childhood or early-onset atopic asthma, (b) adult or late-onset eosinophilic asthma, and (c) aspirin-exacerbated respiratory disease (AERD). A notable distinction lies in the degree of tissue eosinophilia observed in these phenotypes. Early-life or childhood asthma typically displays a tissue eosinophilia rate of about 36%, in contrast to the 63% eosinophil level commonly seen in late-onset asthma [30].

### 3.1. Childhood or Early-Onset Atopic Asthma

Childhood or early-onset atopic asthma typically begins during infancy and is triggered by various factors, including cigarette smoke, allergens, pollutants, atopy, eosinophilia, and rhinovirus-induced wheezing. These triggers activate immune and inflammatory cascades, leading to bronchoconstriction [31,32]. This form of asthma can persist throughout life, posing risks of compromised lung function, long-term health issues, and exacerbated asthma during school age, adolescence, and early adulthood [33]. Genetic factors are thought to influence the immune response, contributing to the development of early-onset asthma [34].

The early-onset asthma phenotype is strongly correlated with the type-2-high subtype of allergic inflammation, characterized by specific biomarkers [35]. Patients with this phenotype typically have circulating allergen-specific IgE antibodies, and their Th2 lymphocytes drive the type 2 immune response by producing the primary Th2 cytokines, such as IL-4, IL-5, and IL-13 [36,37]. IL-5 stimulation is particularly important, as it indicates the presence of sputum and peripheral blood eosinophilia, essential for eosinophil development. Upon re-exposure to triggering allergens, IgE cross-linking occurs, activating the basophils and mast cells. This activation results in the release of significant quantities of histamines, leukotrienes, and prostaglandins, explaining why patients with atopic asthma are susceptible to symptoms of allergic disorders like dermatitis and rhinitis. 

### 3.2. Adult or Late-Onset Eosinophilic Asthma

Adult or late-onset eosinophilic asthma, distinct from early-onset asthma, is characterized by type-2-high eosinophilic airway inflammation that persists even after inhaled corticosteroid treatment [38,39,40]. From its onset, managing its symptoms is challenging, with patients frequently experiencing continued respiratory discomfort and intense exacerbations that may require oral corticosteroid intervention. This phenotype is commonly associated with chronic conditions such as nasal polyposis and rhinosinusitis, which can occur with or without aspirin sensitivity [37]. The development of this type of eosinophilic asthma may be linked to the activation of the innate lymphoid cells, leading to an increase in IL-5 and IL-13 production and elevated fractional exhaled nitric oxide (FeNO) levels [41,42]. Consequently, late-onset eosinophilic asthma often requires specialized therapeutic strategies due to its resistance to standard treatments and its association with chronic rhinosinusitis and persistent eosinophilic inflammation.

### 3.3. Aspirin-Exacerbated Respiratory Disease (AERD)

Aspirin-exacerbated respiratory disease (AERD) represents a significant adult-onset eosinophilic asthma phenotype, characterized by the co-occurrence of eosinophilic asthma with chronic eosinophilic rhinosinusitis, nasal polyps, and heightened sensitivity to COX-1 inhibitors, particularly aspirin. AERD affects approximately 7–8% of all asthma patients and about 15% of those with severe asthma. It typically manifests in young adulthood, initially presenting as sinusitis and rhinitis, and progresses to eosinophilic asthma, nasal polyp development, and increased sensitivity to aspirin and similar medications.

The precise mechanism underlying AERD is not fully understood, but its development is thought to be associated with genetic variations in arachidonic acid (AA) metabolism and the synthesis of cysteinyl leukotrienes (cysLTRs), notably LTC4, D4, and E4. Concurrently, there is a reduction in the expression of prostaglandins (PGE2) and theirEP2 receptor [43,44]. PGE2 plays an essential role in inhibiting the activation of immune cells such as ILC2s, mast cells, and eosinophils. This results in intense eosinophilic inflammation, leading to severe sinusitis and asthma symptoms. Before the advent of biologics targeting IL4Rα, IL5/IL5Rα, and IgE, aspirin desensitization was a critical treatment strategy for AERD [45,46,47].

## 4. Endotypes of Eosinophilic Asthma

Based on the composition of the inflammatory cells and the mediators involved, such as T helper 2 (Th2) cells and type 2 cytokines, two primary endotypes of severe asthma have been identified: Th2-high eosinophilic asthma and Th2-low (or non-Th2) non-eosinophilic asthma (neutrophilic asthma). This distinction is crucial for guiding targeted treatment strategies and understanding the underlying mechanisms of different asthma presentations [48].

At least half of asthma patients have Th2-high eosinophilic asthma, which involves the T helper (Th2) lymphocytes, ILC2s (type 2innate lymphoid cells), and mast cells. This endotype is characterized by the release of specific cytokines and distinct immunoglobulin (Ig) E and mediators and exhibits more severe clinical manifestations, including eosinophilic cell infiltration of the bronchial wall, lower FEV1 values, increased bronchial hypersensitivity, increased use of oral corticosteroids (OCSs), elevated emergency department admissions, and more frequent asthma attacks. Noninvasive biomarkers such as augmented blood and sputum eosinophil counts, increased serum-specific IgE levels, and elevated FeNO levels can indicate the presence of Th2-high eosinophilic asthma [49].

Based on the presence or absence of atopic reactivity, Th2-high eosinophilic asthma can be further categorized into atopic eosinophilic asthma (arising from specific allergic atopic reactivity) and nonatopic eosinophilic asthma (arising from non-specific, non-allergic, nonatopic mechanisms, involving innate immunity). Atopic eosinophilic asthma is driven by high Th2, while nonatopic eosinophilic asthma or innate immune reactions are guided by the type 2 innate lymphoid cells (ILC2s).

Atopic eosinophilic asthma typically begins in childhood and may persist into adulthood, especially in individuals with year-round allergen sensitivity. In atopic eosinophilic asthmatics, exposure to allergens triggers the release of Th2-dependent cytokines (e.g., IL-4, IL-5, IL-13, IL-25, IL-33) and TSL from the airway epithelial cells [50]. Central players in this endotype are the Th2 helper CD4+ cells, basophils, B cells, and mast cells. Within atopic eosinophilic asthma, IL-5 spearheads eosinophil stimulation, differentiation, and survival, while IL-4 and IL-13 are essential for prompting the B cells to produce IgE [51]. Atopic eosinophilic asthmatics are characterized by the presence of specific IgE antibodies, indicated by serum immunoassay results of at least 0.70 KU/L and/or positive skin prick tests (SPT) with papules ≥ 3 mm [52]. A positive correlation exists between elevated total IgE levels and both asthma-related hospitalizations and the need for higher doses of ICS. When high doses of ICS and LABA treatment are inadequate for controlling atopic eosinophilic asthma, omalizumab is a preferred option, necessitating the use of a biologic agent.

Nonatopic eosinophilic asthma exhibits eosinophilia and typically manifests in individuals aged 40 to 50. Such patients experience recurrent asthma attacks with a frequent need for systemic steroids and display negative results in atopy tests, including the skin prick test (SPT) and specific IgE tests. It may also involve sinusitis, nasal polyps, and sensitivity to nonsteroidal anti-inflammatory drugs (NSAIDs) in some cases. In these patients, eosinophilic inflammation is driven by ILC2, which produces IL-5 and IL-13 in response to alarmins (like IL-25, IL-33, and TSLP) from the epithelial cells, independent of allergen exposure [53]. The current therapeutic strategies for Th2-high eosinophilic asthma primarily target eosinophilic interleukins (TSLP, IL-13, IL-5, IL-4) and IgE. However, selecting the most effective agent is complex due to the diverse nature of the endotypes. The distinction between atopic eosinophilic asthma and nonatopic eosinophilic asthma is depicted in Table 1.

**Figure 2:**
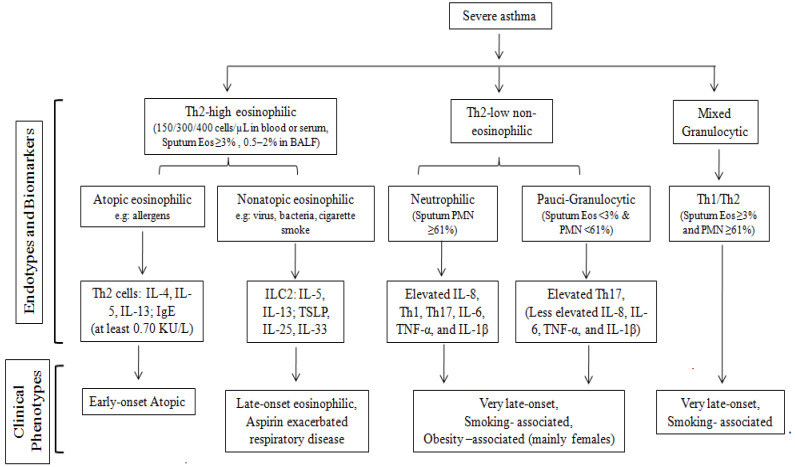
Flow chart showing the endotypes underlying asthma phenotypes.

The other half of asthmatics display Th2-low (or non-Th2) non-eosinophilic asthma, characterized by the involvement of the Th1 and Th17 cells [50,51]. Unlike Th2-high asthma, the Th2-low or neutrophilic asthma endotype includes diverse phenotypes like obesity, smoking, occupational exposures, and aging (usually starting above 50 or 65 years depending on the study). This variant exhibits pronounced remodeling and often resists anti-inflammatory treatment. The Th2-low asthma category encompasses two sub-endotypes: neutrophilic asthma (characterized by neutrophilic airway inflammation) and paucigranulocytic asthma (defined by minimal granulocyte presence, where both eosinophilic and neutrophilic inflammation coexist) [54]. A subset of patients demonstrates characteristics of both Th2-high and Th2-low endotypes, featuring the presence of both neutrophils and eosinophils, commonly known as Mixed Granulocytic Endotypes. Identifying the optimal treatment approach to cases involving mixed endotypes and Th2-low asthma can be particularly challenging, as it requires a more nuanced understanding of the underlying immune mechanisms at play. Figure 2 describes various endotypes, including eosinophilic and non-eosinophilic asthma.

## 5. Diagnostic Biomarkers for Eosinophilic Asthma

### 5.1. Eosinophil as a Biomarker

#### 5.1.1. Sputum Eosinophil Count

Airway inflammation in eosinophilic asthma can be indicated by sputum eosinophilia, determined by an eosinophil count that surpasses 2–3% of the total cells in sputum samples [55]. Corticosteroids and specific biological agents have demonstrated positive results in mitigating sputum eosinophilia [56,57,58,59], while drugs targeting IL-13 have shown inconsistent outcomes [60,61]. Due to the intricate and time-intensive nature of sputum induction and quantification, researchers are increasingly pivoting toward alternative diagnostic biomarkers associated with eosinophilic inflammation.

#### 5.1.2. Blood/Serum Eosinophil Count

Eosinophilic asthma is often indicated by peripheral blood eosinophil counts that surpass specific benchmarks, such as >150 cells/μL, >300 cells/μL, or >400 cells/μL. These elevated counts are frequently linked to the severity of asthma exacerbations [62,63]. Interestingly, there is a discernible correlation between eosinophil counts in the sputum and blood. Treatments like corticosteroids [64] and various biological agents [15,65,66,67,68] have been observed to reduce blood eosinophilia.

Elevated levels of eosinophil-derived neurotoxin (EDN), a substance released from the eosinophils, indicate the activation of an eosinophilic biomarker in eosinophilic asthmatics [69]. Benralizumab has been shown to reduce EDN levels [68], suggesting that EDN could serve as a response biomarker [70] and indicate the extent of eosinophilic airway inflammation [69].

Eosinophil peroxidase (EPO) is released by the eosinophils according to an IgE-dependent mechanism. The serum levels of EPO have been found to be higher in asthmatics compared to healthy individuals [71], suggesting that EPO could serve as a biomarker for eosinophilic activation. Additionally, an increased risk of exacerbations and severe airflow limitation has been observed in severe asthmatics with persistently high sputum EPO levels [72].

#### 5.1.3. Bronchoalveolar Lavage Fluid (BALF) and Biopsy of the Airway Mucosa 

Beyond sputum and blood/serum eosinophil counts, assessments can also be made according to the examination of the eosinophils in the bronchoalveolar lavage fluid (BALF) or via biopsy of the airway mucosa. In BALF, the most commonly cited cut-off level is 0.5–2% of all analyzed cells, while in bronchial biopsy samples, the range is 5–20 cells/mm^2^. The suggested cut-off levels for the absolute number of eosinophils in asthmatics are outlined in Table 2.

### 5.2. Fraction of Exhaled Nitric Oxide (FeNO)

Elevated fractional exhaled nitric oxide (FeNO) levels, particularly above 25 parts per billion (ppb), are indicative of eosinophilic airway inflammation and can predict responsiveness to corticosteroids [73]. Several studies have underscored the importance of FeNO in assessing airway inflammation and in evaluating the effectiveness of treatment [74,75,76,77]. Recent insights suggest that combining FeNO levels with blood eosinophil counts can further optimize the management of asthma [78].

### 5.3. Exhaled Breath Condensate (EBC)

Exhaled Breath Condensate (EBC) offers a noninvasive method for assessing severe eosinophilic asthma. It includes compounds such as cysteinyl leukotrienes, which have been found to correlate with asthma exacerbations [79]. Recent advancements in this field have highlighted the potential of metabolomic analysis of EBC in asthma research, opening new avenues for understanding and managing the disease.

### 5.4. Urinary Biomarkers

The progression of eosinophilic asthma results in significant changes in the composition of urine metabolites [80,81,82]. Key urinary markers, such as bromotyrosine, have been identified as valuable indicators of the disease’s trajectory. These markers not only offer insights into the progression of eosinophilic asthma but also serve as important tools for assessing the efficacy of steroid therapy.

### 5.5. OMICS

Recent studies in the field of ‘omics’ have identified three transcriptome-associated clusters (TACs) named TAC1, TAC2, and TAC3. Patients with severe asthma in the TAC1 group exhibit oral corticosteroid dependence, frequent exacerbations, and severe respiratory distress. They show the highest expression of IL-13/Th2 and type 2 innate lymphoid cells (ILC2s), which are associated with increased sputum eosinophilia [83,84]. Unlike TAC1, TAC2 and TAC3 are not associated with Th2 inflammation. In another study, six genetic markers, including non-physiological isozymes (ALPL), Charcot–Leyden crystal protein (CLC), deoxyribonuclease 1-like 3 (DNASE1L3), chemokine receptor 2 (CXCR2), and carboxypeptidase A3 (CPA3), were analyzed. It was found that corticosteroids are associated with a better response in these markers, aiding in the distinction of asthma endotypes [49,85]. Furthermore, genetic markers and proteomic studies of airway tissues are unveiling potential therapeutic targets and elucidating the underlying molecular landscape of asthma [49].

### 5.6. Micro RNAs (miRNAs)

Micro RNAs (miRNAs) play a significant role in Th2-driven airway inflammation in eosinophilic asthma. A wide range of miRNAs, including miR-21, miR-135a, miR-142, miR-143, miR-146b, miR-193b, miR-223, miR-365, miR-375, miR-452, and miR-1165-3p, have been implicated in this process [86]. Profiling these miRNAs can aid in distinguishing severe asthmatic patients from healthy individuals and in predicting responses to treatment [87]. Specifically, analyzing miRNA-338 and miRNA-145 in sputum samples has proven useful in differentiating patients with severe eosinophilic asthma from those with Chronic Obstructive Pulmonary Disease (COPD) [88]. Notably, miRNA-338-3p has been identified as a potential early biomarker for response to reslizumab and mepolizumab treatments in severe eosinophilic asthma [89].

### 5.7. Periostin

Periostin plays a crucial role in tissue remodeling and inflammation and has emerged as a potential prognostic factor for asthma. Despite corticosteroid treatment, elevated serum periostin levels and increased bronchial epithelial cell proliferation are associated with more frequent asthma exacerbations and persistent eosinophilic airway inflammation. Periostin is released in response to stimulation from the IL-4 and IL-13 signaling pathways. Interestingly, therapies targeting IL-4 and IL-13 have been shown to reduce periostin levels. Specific treatments such as omalizumab, Lebrikizumab, and Tralokinumab have been effective in decreasing the periostin levels in the airways, suggesting their potential role in managing asthma-related tissue remodeling and inflammation [90,91].

### 5.8. Serum Immunoglobin E 

Elevated levels of IgE have been noted in atopic eosinophilic asthmatics, particularly those with sputum eosinophilia and airway eosinophilic inflammation. However, IgE is not as strong a biomarker for asthma exacerbations as other eosinophil-related biomarkers like blood eosinophils and fractional exhaled nitric oxide (FeNO). This is because the IgE levels initially rise and then typically return to normal within 1–2 months. Despite this, anti-IgE therapy, notably with omalizumab, has shown significant effectiveness. In patients with moderate to severe persistent allergic asthma, omalizumab treatment rapidly reduces the free IgE levels and FcεRI expression on the basophils and mast cells. It also leads to a decrease in peripheral blood and sputum eosinophil counts [92,93,94].

### 5.9. Future Biomarkers and Limitations

Beyond the established biomarkers in eosinophilic asthma, emerging ones like IL-6 [95], Notch4 [50], and the IL-4Rα-R576 allele [96] show promise in predicting responses to biological therapies. A phase IV randomized placebo-controlled trial (NCT03694158) is currently investigating the impact of dupilumab in patients with the IL-4Rα-R576 allele.

Siglec-8, a surface molecule expressed on eosinophils, may correlate with eosinophilic airway inflammation when measured in sputum or exhaled breath. Markers of eosinophil activation such as CD11b or CD62L could provide real-time information about ongoing inflammation. Additionally, CD69, CX3CR1, B7-2/CD86, and MHC II may serve as prognostic biomarkers due to their increased expression upon eosinophil activation. Elevated IFN-γ/IL-6 gene levels offer insights into asthma, including eosinophilia, phenotypic distinctions, and increased immune cell infiltration in the airway submucosa [97]. 

Volatile organic compounds (VOCs) are emerging as potential biomarkers for asthma, playing a crucial role in differentiating asthma phenotypes, predicting exacerbations, and assessing responsiveness to steroid therapy [98]. Standardization of the collection methods and validation of the analysis techniques are needed for their broader application. Factors like diet, bacteria, and environmental contaminants can influence VOC levels, complicating their diagnostic use.

The elevated mast cell tryptase levels in severe asthma patients suggest its importance, especially when combined with TSLP and blood eosinophil count, in linking to exacerbation risks. Mepolizumab significantly decreases the serum tryptase levels in patients with eosinophilic asthma and idiopathic mast cell activation syndrome [99]. Similarly, IL-13, along with biomarkers like periostin, blood eosinophils, and FeNO, is significantly higher in eosinophilic asthmatics [100]. The chitinase-like protein YKL-40, positively correlated with peripheral blood eosinophils and total serum IgE levels, emerges as another potential biomarker [101].

Proteomic analysis offers a roadmap for identifying potential biomarkers associated with eosinophilic asthma [102]. Imaging biomarkers and AI, in conjunction with cluster analyses, could enhance the effectiveness of models determining responses to specific biologic therapies [103,104].

However, the use of biomarkers in predicting responses to biological treatments has limitations. For example, the skin prick test (SPT) and specific IgE detection tests indicate atopic sensitization but can lead to false positives [105].The biomarkers correlating with the mechanism of action of a biological therapy, like blood eosinophil counts for anti-IL-5 agents, tend to decrease during treatment, limiting their usefulness to pre-treatment only. Additionally, environmental and clinical factors, such as varying blood eosinophil count cut-offs across populations, FeNO levels, and oral corticosteroid use, affect biomarker interpretation [10,106]. The Global Initiative for Asthma 2022 guidelines recommend repeated testing and acknowledges fluctuations and variations in patient populations.

## 6. Therapeutic Approaches

For patients with severe asthma, the first-line treatments typically include oral glucocorticoids, long-acting beta-agonists (LABAs), inhaled corticosteroids (ICSs), and leukotriene receptor antagonists (LTRAs) [107,108,109,110,111,112,113,114,115]. However, challenges arise with the prolonged and high-dose use of systemic corticosteroids due to their potential side effects. Notably, 5 to 10% of severe eosinophilic asthma patients exhibit compromised responsiveness to corticosteroids, leading to either increased dependency or diminished efficacy [116,117,118,119].

Patients with eosinophilic asthma often continue to experience persistent symptoms or recurrent flare-ups even after extensive standard treatments, including oral and inhaled glucocorticoids, LABAs, and long-acting muscarinic antagonists (LAMAs). In cases where conventional treatments are insufficient, particularly for difficult-to-treat or treatment-resistant severe eosinophilic asthma, the focus shifts to specialized biologic therapies. These therapies target specific molecules or inflammation triggers and have shown benefits in reducing asthma episodes, improving lung function, and enhancing the overall asthma management in severe eosinophilic asthmatics.

Several biologic agents specifically designed for severe eosinophilic asthma include IL-5 inhibitors like reslizumab and mepolizumab and the IL-5 receptor antagonist benralizumab. Additionally, non-IL-5-specific biologic therapies such as Lebrikizumab and Tralokinumab (IL-13 inhibitors); dupilumab, which blocks the signaling of both IL-4 and IL-13 by targeting IL-4Rα; and omalizumab, an IgE blocker, are also beneficial. Furthermore, an anti-TSLP molecule (Tezepelumab and Ecleralimab), currently under development, shows promise for individuals with both eosinophilic and non-eosinophilic asthma categories (Figure 1). Comprehensive details about these biologics, including data from randomized clinical trials and ongoing studies, are provided in Table 3, Table 4 and Table 5.

The safety profiles of the currently approved biologics for eosinophilic asthma are generally encouraging, with a good tolerance overall. Common reactions, such as those at the injection site, are observed with all biologics but rarely necessitate treatment discontinuation. However, the specific side effects and safety concerns vary among these agents. Hypersensitivity reactions are infrequent across all approved biologics. Omalizumab and mepolizumab, in rare cases, are associated with anaphylaxis and zoster infections, respectively, with an epinephrine autoinjector recommended for omalizumab users. Dupilumab therapy may be accompanied by helminthic infections and transient eosinophilia. Agent-specific adverse reactions, both common and rare, have been summarized in Table 3.

**Figure 3:**
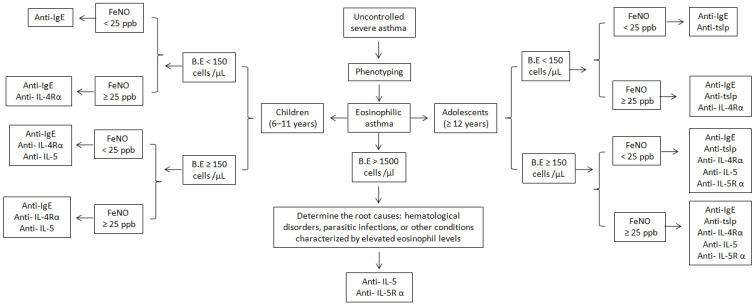
Algorithm for selection of an appropriate biological drug in children and adolescents with eosinophilic asthma. Omalizumab is exclusively considered for atopic eosinophilic asthma, whereas all other biological drugs are applicable to both atopic and nonatopic forms of eosinophilic asthma.

The selection of appropriate biologic medication for eosinophilic asthma is based on patient age and biomarker levels, focusing on phenotypic characterization, including blood eosinophil counts and FENO levels. This selection process is depicted in the algorithm presented in Figure 3. The absence of direct head-to-head comparison trials for biologics and the lack of conclusive evidence to rank specific biologics in order of efficacy complicates the decision-making process.

While biologics effectively reduce the severity and rate of exacerbations, their impact on lung function improvement varies. In cases of significantly compromised lung function, biologics that specifically enhance lung function (e.g., dupilumab) should be preferred over those with modest effects on lung function (e.g., omalizumab and mepolizumab). Additionally, the presence of comorbid conditions that may benefit from specific biologics should be considered. For instance, dupilumab is expected to improve eosinophilic esophagitis alongside eosinophilic asthma, and omalizumab is effective for chronic spontaneous urticaria and allergic asthma.

A collaborative decision-making process involving the patient and their family is beneficial during biologic selection, as individual preferences, perspectives, and goals vary. Factors such as the route of administration and frequency and number of injections may influence a patient’s decision and treatment adherence. An open conversation about the aspects and characteristics of biologics with patients and their families ideally leads to maximal treatment adherence and optimal outcomes [120].

Close clinical follow-up is essential to evaluate a patient’s response to biologic therapy. Although universal criteria for a favorable outcome are absent, key indicators include diminished asthma episodes, symptom improvement, and enhanced quality of life. FENO and lung function improvements may be observed within 2 to 4 weeks after initiating therapy, while monitoring for exacerbations should continue for 4 to 6 months. The occurrence of an exacerbation after starting biologic therapy should not be immediately considered a treatment failure, as biologics typically do not completely eliminate exacerbations. For patients showing a partial response within 4 to 6 months, extending the trial or switching to a different biologic targeting a different pathway may be considered. Prior to switching, a thorough reassessment of asthma indicators and biomarkers is advocated. The concurrent use of dual biologic therapies is generally discouraged due to financial constraints and limited empirical evidence.

The real-world data suggest that all biologics are effective at reducing the frequency of severe asthma exacerbations, improving lung function, and reducing the need for oral corticosteroids. Specifically, dupilumab has shown significant improvements in lung function and a reduction in exacerbation rates of 60–70%, as detailed in Table 6.

Clinicians bear the responsibility of making decisions grounded in diverse factors, including a patient’s lung function, asthma history, oral glucocorticoid usage, key biomarkers (blood eosinophil count and FeNO), and overall quality of life. Ancillary considerations, such as the age of asthma onset, comorbid health conditions, the drug administration mode, the dosing frequencies, healthcare monitoring needs, insurance policies, patient preferences, and the associated costs, are crucial in selecting a specific biologic therapy.

**Table 3:** Detailed description of biologics for eosinophilic asthma.

Monoclonal Antibody	Target	Mechanism of Action	Patient Selection/Clinical Indications	Response Criteria	Dosing	Adverse Effect/Safety Concerns	Efficacy
Reslizumab	IL-5	Binds to IL-5, inhibiting the maturation, activation, survival, migration, and entry of eosinophils into the airways	Patients with severe eosinophilic asthma who have uncontrolled symptoms despite medium- to high-dose ICS and LABA therapy	Eosinophil count > 400 cells/µL, ≥2 asthma exacerbations in the last year, OCS dependence, comorbidity with nasal polyposis	≥18 y: 3 mg/kg IV monthly -Home administration allowed	-Reactions at the injection site * -HES (EGPA) ** -Hypersensitivity reactions ***	-Improves lung function -Reduces exacerbations and asthma symptoms
Mepolizumab	IL-5	Binds to IL-5, preventing its binding to the IL-5 receptor and causing apoptosis of eosinophils	Patients with severe eosinophilic asthma who have uncontrolled symptoms despite moderate/high-dose ICS and LABA therapy	Peripheral blood eosinophil counts ≥ 150 cells/µL at the beginning of treatment or ≥300 cells/µL in the last year, ≥2 asthma attacks in the last year, presence of nasal polyposis, and OCS dependence	6–11 y: 40 mg SC monthly ≥12 y: 75–100 mg SC monthly -Home administration allowed	-Reactions at the injection site * -HES (EGPA) ** -Hypersensitivity reactions, herpes zoster infection ***	-Improves asthma control and lung function -Reduces blood eosinophilia, severe exacerbations, and corticosteroid usage
Benralizumab	IL-5Rα	Binds to IL-5Rα, preventing IL-5 binding to the receptor and causing rapid apoptosis of eosinophils through antibody-dependent cytotoxicity	Patients with uncontrolled severe eosinophilic asthma, despite the use of high-dose ICS and LABA therapy	Patients with higher exacerbations and baseline blood eosinophil counts	≥12 y: 30 mg SC every 4 or 8 weeks -Home administration allowed	-Reactions at the injection site, nasopharyngitis * -Helminthic infection ** -Hypersensitivity reactions ***	-Improves quality of life and lung function -Reduces oral corticoid use and exacerbations
Dupilumab	IL-4Rα	Binds to IL-4Rα and inhibits the downstream signaling of both IL-4 and IL-13	Patients with uncontrolled severe asthma who experience two or more moderate or severe asthma exacerbations per year	Blood eosinophil count ≥ 300 cells/µL, FEV1 < 1.75 L, and elevated FeNO levels	6–11 y (≤30 kg): 100 mg SC every 2 weeks or 300 mg SC monthly 6–11 y (>30 kg): 200 mg SC every 2 week ≥12 y: 200 mg or 300 mg SC every 2 week -Home administration allowed	-Reactions at the injection site * -Transient eosinophilia, helminthic infection ** -Hypersensitivity reactions ***	-Improves quality of life and lung function -Reduces severe exacerbations, oral corticosteroid use
Tezepelumab	TSLP	Inhibits TSLP-induced signaling	Patients with severe asthmatics	Patients with basal blood eosinophils ≥ 150 cells per µL	6–11 y: 70 mg SC monthly ≥12 y: 210 mg SC monthly -Home administration not allowed	-Reactions at the injection site, nasopharyngitis * -Pharyngitis, arthralgia, back pain ** -Hypersensitivity reactions ***	-Improves lung function and quality of life -Reduces asthma exacerbations
Omalizumab	IgE	-Binds to the constant region of free IgE, preventing its interaction with IgE receptors -Inhibits mast cell activation by FcεRI -Inhibits FcεRII on eosinophils and dendritic cells	Severe allergic asthmatics who are sensitive to at least one perennial allergen and have uncontrolled symptoms despite medium-tohigh-dose ICS and LABA therapy	-Patients with blood eosinophil ≥ 260 cells/µL and FeNO ≥ 20 ppb	≥6 y: 75–375 mg SC every 2–4 week (Based on total IgE& weight) -Home administration allowed	-Reactions at the injection site * -HES (EGPA), serum sickness ** -Anaphylaxis (black box warning) ***	-Improves quality of life -Reduces exacerbations and corticosteroid usage

* Common adverse reactions, ** agent-specific adverse reactions, *** rare agent-specific adverse reactions. y, year; SC: subcutaneous; HES: hyper-eosinophilic syndrome; EGPA: eosinophilic granulomatosis with polyangiitis.

**Table 4:** Completed randomized clinical trials of biological for eosinophilic asthma.

Target	Drug	Clinical Trial No	Patients	Dose and Duration	Outcome	Ref.
IL-5	Mepolizumab	NCT01000506 DREAM	621	75–750 mg IV 52 W	-Reduced PB and SP eosinophils -48% reduction in exacerbations at 52 W	[121]
		NCT01691521 MENSA	576	75 mg IV: 100 mg SC 32 W	-53% reduction in AAER at 32 W -Improved FEV1, PEF, SGRQ, and ACQ at 32 W	[122]
		ISRCTN75169762	61	750 mg IV 52 W	-57% reduction in AAER at 50 W -Reduced PB and SP eosinophils	[15]
		NCT00292877	9	750 mg IV 26 W	-Reduced AAER, SP, and PB eosinophils -Improved FEV1 and ACQ	[56]
		NCT01691508	135	100 mg SC 20 W	-Significant glucocorticoid-sparing effect -Reduced exacerbations -Improved control of asthma symptoms	[123]
		NCT01691859	347	100 mg SC 52 W	-61% reduction in AAER -Improved FEV1 and ACQ-5 at 24 W	[124]
		NCT02135692 COSMEX	339	100 mg SC 172 W	-Reduced AAER and PB eosinophils -Improved FEV1	[125]
			145	100 mg SC 32 W	-64% reduction in exacerbations -Reduced PB and SP eosinophils	[126]
		NCT02281318 MUSCA	274	100 mg IV 52 W	-58% reduction in AAER at 24 W -Improved FEV1 at 24 W	[127]
		NCT01842607	558	100 mg SC 52 W	-Reduced exacerbation rates	[128]
	Reslizumab		53	3 mg IV 15 W	-Improved FEV1 -Reduced sputum eosinophils	[57]
		NCT01508936	492	3 mg IV 16 W	-Improved FEV1 -Reduced PB eosinophils	[129]
		NCT01287039 NCT01285323	128 104	3 mg IV 52 W	-Reduced AAER	[65]
		NCT01270464	315	0.3–3 mg IV 16 W	-Improved ACQ, AQLQ, FEV 1,and FVC	[66]
			1051	3 mg IV 24 months	-Improved ACQ, FEV 1, and FVC -Reduced PB eosinophils	[130]
		NCT01287039 /NCT01285323	273	3 mg IV 52 W	-Reduced AAER and ACQ	[131]
		NCT02452190 NCT02501629		110 mg SC 52 W	-Fixed dose (110 mg) was ineffective in reducing exacerbation rates and blood eosinophil count	[132]
IL-5Rα	Benralizumab	NCT01238861	324	2–100 mg SC one year	-Reduced blood eosinophils count and asthma exacerbations	[133]
		NCT01928771 SIROCCO	1205	30 mg SC 48 W	-Reduced AAER and PB eosinophils -Improved FEV 1 and ACQ-6	[134]
		NCT01914757 CALIMA	1306	30 mg SC 56 W	-Reduced PB eosinophils and AAER -Improved ACQ and FEV 1	[135]
		NCT02075255	369	30 mg SC 28 W	-Improved AAER, ACQ-6, and AQLQ	[136]
			18	30 mg SC 28 W	-Reduced BP and SP eosinophils	[137]
		NCT00768079	74	0.3–1 mg SC 24 W	-Reduced PB eosinophils and AAER	[138]
		NCT02322775.	351	30 mg SC 12 W	-Increased FEV 1 at 12 W	[139]
1L-13	Lebrikizumab	NCT00930163	219	250 mg SC 24 W	-Reduced exacerbation and FeNO -Improved FEV1	[140]
		NCT01867125 NCT01868061	1081	5–125 mg SC 52 W	-Reduced exacerbation but not significant	[141]
	Tralokinumab	NCT01402986	452	300 mg SC 52 W	-Improved FEV1	[142]
		NCT02161757 NCT02194699	398	300 mg SC 52 W	-Reduced AAER in FeNO-high patients	[143]
		NCT02449473 MESOS	79	300 mg SC 12 W	-Did not reduce eosinophils, FENO, and IgE	[60]
IL-4Rα	Dupilumab	NCT01312961	52	300 mg SC 12 W	-Reduced exacerbation and FeNO -Improved FEV 1 and ACQ	[144]
		NCT01854047	769	200–300 mg SC 24 W	-Reduced exacerbation and FeNO -Improved FEV 1 and ACQ	[145]
		NCT02414854	1902	200–300 mg SC 52 W	-Reduced AAER and FeNO -Improved FEV 1 and ACQ	[146]
		NCT02528214	210	300 mg SC 24 W	-Reduced exacerbation -Improved FEV 1	[147]
IgE	Omalizumab	NCT01922037	806	450 mg 48 W	-Improved exacerbation rates -Reduced hospitalizations	[148]
		NCT00314574	850	0.016 mg SC 48 W	-Reduced AAER and FeNO -Improved FEV 1	[149]
			45	0.016 mg IgE (IU/mL) SC 16 W	-Reduced SP, submucosal and epithelial eosinophils -Reduced serum IgE	[150]
TSLP	Tezepelumab	NCT02054130 PATHWAY	549	70–280 mg SC 52 W	-Reduced blood eosinophil counts, Feno and total serum IgE levels	[67,151]
		NCT01405963	31	700 mg SC 12 W	-Reduced eosinophils -Improved FEV 1 and FeNO l	[152]
		NCT03347279	1061	210 mg SC 52 W	-Reduced AAER, PB eosinophils, FeNO and serum IgE -Improved ACQ	[153]
		NCT04048343 NOZOMI	65	210 mg SC 52 W	-Reduced exacerbation rates -Improved lung function	[154]
ST2	Astegolimab	NCT02918019	502	70–490 mg SC 54 W	-Reduced AER and eosinophils count	[155]
IL-33	Itepekimab	NCT03387852	296	300 mg SC 12 W	-Improved lung function -Reduced eosinophils count	[156]
GATA3	SB010	NCT01743768	40	10 mg SC 4 W	-Reduced FEV1, sputum eosinophilia, and FeNO	[157]

Abbreviations: PB, peripheral blood; SP, sputum; FEV1, forced expiratory volume (amount of air that a person can force out of their lungs in 1 s); AAER, annualized asthma exacerbation rate; SC, subcutaneous; IV, intravenous; FeNO, fractional exhaled nitric oxide; W, weeks; ACQ, asthma control questionnaire; AQLQ, asthma quality of life questionnaire; SGRQ, St George’s Respiratory Questionnaire.

**Table 5:** Ongoing clinical trials of biologic therapies for eosinophilic asthma.

Target	Drug	Phase	ClinicalTrials.gov Identifier	Status
IL-5	Mepolizumab	IV	NCT03476109	Recruiting
		IV	NCT05626777	Recruiting
		IV	NCT04276233	Recruiting
		III	NCT04680611	Recruiting
		III	NCT02594332	Terminated (recruitment problem)
		III	NCT04607005	Active, not recruiting
		III	NCT04718389	Recruiting
		II	NCT04641741	Recruiting
			NCT05144087	Not recruiting
			NCT04612556	Active, not recruiting
			NCT05040997	Recruiting
			NCT05241769	Active, not recruiting
			NCT05689931	Recruiting
			NCT05189613	Recruiting
			NCT05063981	Recruiting
			NCT05404763	Active, not recruiting
	Depemokimab	III	NCT04718389	Recruiting
		III	NCT05243680	Recruiting
		III	NCT04718103	Active, not recruiting
		III	NCT04719832	Active, not recruiting
		III	NCT05274750	Recruiting
		III	NCT05281523	Recruiting
		I	NCT05602025	Active, not recruiting
	Reslizumab	IV	NCT04710134	Active, not recruiting
		III	NCT03052725	Active, not recruiting
		III	NCT01290887	Terminated (business decision)
		IV	NCT02937168	Terminated (business decision)
			NCT04612556	Active, not recruiting
IL-5Rɑ	Benralizumab	IV	NCT03953300	Recruiting
		III	NCT05692180	recruiting
		III	NCT04718389	recruiting
			NCT05440656	Recruitinga
			NCT04221802	Active, not recruiting
			NCT05078281	Recruiting
1L-33	Etokimab	II	NCT03469934	Completed
	Tozorakimab	II	NCT04570657	Active, not recruiting
IL-4Rα	Elarekibep	II	NCT04643158	Recruiting
		II	NCT05794672	Not recruiting
	CM310	II	NCT05186909	Recruiting
		III/II	NCT05761028	Not recruiting
	Dupilumab	II	NCT05347771	Recruiting
		IV	NCT04203797	Active, not recruiting
		IV	NCT03694158	Recruiting
		III	NCT03560466	Active, not recruiting
		IV	NCT04502862	Active, not recruiting
		II	NCT05720325	Recruiting
			NCT05478824	Recruiting
		II	NCT05575037	Active, not recruiting
IgE	Omalizumab	IV	NCT03476109	Recruiting
		IV	NCT04763447	Recruiting
		II	NCT00162773	Active, not recruiting
			NCT05972213	Active, not recruiting
TSLP	Tezepelumab	IV	NCT05329194	Recruiting
		III	NCT03927157	Recruiting
		III	NCT05280418	Recruiting
		III	NCT05398263	Recruiting
		III	NCT05274815	Recruiting
	Ecleralimab	II	NCT04410523	Terminated (sponsor decision)
		II	NCT04946318	Terminated (sponsor decision)
	AZD8630	I	NCT05110976	Completed
mIgE on B cells	FB825	II	NCT05008965	Recruiting
Tryptase	Rilzabrutinib	II	NCT05104892	Recruiting
Tyrosine kinase, PDGFR	Masitinib	III	NCT03771040	Recruiting

Abbreviation: IgE, Immunoglobulin E; mIgE, membrane-bound IgE; PDGFR, platelet-derived growth factor receptor; TSLP, thymic stromal lymphopoietin.

**Table 6:** Real-world effectiveness of biologics.

Biologics	Prediction Factors	Exacerbation Reduction	Lung Function Improvement	Oral Corticosteroid Reduction
Dupilumab	Eosinophilic phenotype, blood eosinophil count, FeNO level	60–70%	Significant	Significant
Mepolizumab	Eosinophilic phenotype, blood eosinophil count, FeNO level	50–60%	Variable	Significant
Benralizumab	Eosinophilic or allergic phenotype, blood eosinophil count, FeNO level	50–60%	Variable	Significant
Tezepelumab	Eosinophilic or allergic phenotype, blood eosinophil count, FeNO level	50–60%	Variable	Significant
Omalizumab	Allergic phenotype, blood eosinophil count, FeNO level	50–60%	Variable	Significant

### 6.1. Possible Potential Therapeutic Targets from Clinical and Pre-Clinical Studies

The current research on monoclonal antibodies (mAbs) is focused on identifying novel therapeutic targets, particularly targeting cytokines and alarmins [158,159]. Astegolamib, an anti-SAT2 or IL-33 receptor antagonist, has been found to reduce the asthma exacerbation rate in eosinophilic asthmatics [155]. Itepekimab, a monoclonal antibody against IL-33, improved lung function in this patient group [156]. Clinical trials are ongoing for Tozorakimab, a human IgG1 anti-IL-33 antibody. IL-25, an epithelial-derived alarmin also known as IL-17E, is emerging as a promising therapeutic target for asthma, especially in patients with eosinophilic asthma [160]. Interleukin-23, which mediates eosinophil infiltration and the production of both type 17 and type 2 helper T (Th2) cytokines, is another area of interest. However, Risankizumab, an interleukin-23 inhibitor, has shown underwhelming results [161], indicating the need for further research.

CRTh2, a prostaglandin D2 receptor found in allergic cells like eosinophils, mast cells, and basophils, is another potential target. PGD2 receptor antagonists, such as Fevipiprant [59,162] and OC000459 (antagonists of CRTh2) [163], have proven effective in reducing eosinophilic airway inflammation in persistent eosinophilic asthma. Multiple studies on Fevipiprant are currently in progress. Additionally, TL1A (tumor necrosis factor-like cytokine 1A) blockade, as seen with C03V (anti-TL1A), may be effective in reducing inflammation and cytokine levels in eosinophilic asthma [164].

Beyond biological therapies, other potential therapeutic options for eosinophilic asthma include Siglec-8, p38 inhibitors, CDK inhibitors, NF-κB inhibitors, HDACs, PI3K inhibitors, MLCK inhibitors, nitric oxide (NO)-donating compounds and ADAM8. These agents could inhibit eosinophil survival, proliferation, and migration and reduce airway inflammation. However, more research is required to assess their efficacy and safety in clinical trials.

#### 6.1.1. Siglec-8

Siglec-8, a protein expressed predominantly on human eosinophils, mast cells, and, to a lesser extent, basophils, represents a promising target in eosinophilic asthma. Preclinical studies have highlighted its role in inhibiting mast cell activity and reducing eosinophil levels [165,166]. Lirentelimab (AK002), a humanized antibody that targets Siglec-8, is currently under clinical development for conditions like eosinophilic gastritis and duodenitis [167], as well as eosinophilic esophagitis (clinical trial NCT04322708). Given its specific action on cells implicated in eosinophilic asthma, Siglec-8 is emerging as a potential therapeutic agent for this disease.

#### 6.1.2. P38 MAPK (Mitogen-Activated Protein Kinase)

Mitogen-activated protein kinases (MAPKs), which include the p38, ERK, and JNK subtypes, play crucial roles in a variety of cellular processes. These kinases phosphorylate transcription factors, leading to gene transcription, and are involved in cell apoptosis, survival, development, proliferation, and differentiation [168]. Specifically, the p38 MAPK has a significant influence on eosinophil survival. Inhibitors targeting p38 promote eosinophil apoptosis and have been shown to alleviate uncontrolled eosinophilia [169]. Notably, in mouse models of asthma, p38 inhibitors have demonstrated potential in reducing allergen-induced pulmonary eosinophilia, airway hyperresponsiveness, and mucus hypersecretion. This suggests that targeting the p38 MAPK pathway could be a promising therapeutic approach in the management of eosinophilic asthma [170].

#### 6.1.3. Cyclin-Dependent Kinases (CDKs)

Cyclin-dependent kinases (CDKs) are crucial enzymes that play a key role in cell cycle regulation by phosphorylating transcription factors and tumor suppressor proteins. Eosinophils express several CDKs, including CDKs 1, 2, 5, 7, and 9. Selective inhibitors of these kinases, such as AT7519 and R-roscovitine, have demonstrated the ability to promote spontaneous and IL-5-induced apoptosis in vitro [171,172]. This suggests a potential therapeutic role of CDK inhibitors in eosinophilic asthma. However, the effectiveness of these inhibitors in treating eosinophilic asthma disorders in clinical settings remains to be evaluated using clinical trials.

#### 6.1.4. NF-κB

NF-κB, predominantly found in inflammatory cells, plays a pivotal role in the pathogenesis of asthma, characterized by increased expression and transcriptional activity. Its constitutive activity is crucial for the persistence of eosinophils, and inhibiting NF-κB function has been shown to significantly increase eosinophil apoptosis [173,174,175]. This finding has led researchers to explore various inhibitors targeting NF-κB. These include small interfering RNA (siRNA), proteasome inhibitors, and small molecule inhibitors of IκB kinase-β. These inhibitors have been investigated in animal models of asthma, offering potential avenues for novel therapeutic strategies in the management of eosinophilic asthma.

#### 6.1.5. Histone Deacetylases (HDACs)

Inhibiting histone deacetylases (HDACs) results in the dissociation of histone–DNA interactions, leading to increased gene expression [176]. HDAC inhibitors, such as trichostatin A (TSA) and apicidin, have shown potential in reducing inflammation. They achieve this by promoting apoptosis in eosinophils and neutrophils [177], suggesting their anti-inflammatory effects [178]. However, the application of HDAC inhibitors in animal models of asthma, especially in the context of eosinophilic inflammation, has yielded mixed and inconclusive results. Studies indicate that TSA can reduce methacholine-induced airway hyperresponsiveness while either maintaining or decreasing the eosinophil counts in the bronchoalveolar lavage fluid (BALF) [179,180]. These findings underline the potential of HDAC inhibitors as therapeutic agents in eosinophilic asthma, although more research is needed to fully understand their efficacy and mechanism of action.

#### 6.1.6. PI3K

Selective (IC87114) and non-selective (LY294002 and wortmannin) inhibitors of phosphoinositide 3-kinase (PI3K) have shown promise in attenuating Th2 cytokine production, tissue eosinophilia, airway mucus production, and hyperresponsiveness in asthma [181]. The inhibition of PI3K has been demonstrated to reduce the persistence of eosinophils induced by IL-5 [182,183] and plays a significant role in controlling eosinophil migration, which, in turn, influences their survival [181]. Given these effects, targeting PI3K may represent a promising approach to the therapeutic management of asthma, particularly for strategies aimed at reducing eosinophil-driven inflammation.

#### 6.1.7. MLCK (Myosin Light Chain Kinase)

Myosin light chain kinase (MLCK) is implicated in the autoimmune reactions associated with asthma. Its expression is linked to several key processes in the disease, including eosinophil accumulation, the release of various cytokines, mucus production, the exacerbation of airway inflammation, lung remodeling, IgE synthesis, and the progression of asthma [184,185,186]. Inhibiting MLCK has shown therapeutic potential in reducing lung inflammation and remodeling. Given these effects, targeting MLCK presents a viable treatment option for asthma, with the potential to mitigate several key aspects of the disease’s pathology [187,188,189].

#### 6.1.8. Nitric Oxide (NO)

Nitric Oxide (NO) has been recognized for its anti-eosinophilic properties in the context of asthma [190,191,192], suggesting its therapeutic potential. Compounds like orazipone and its derivatives, including OR-1958 and OR-2370, as well as levosimendan, have demonstrated the ability to diminish lung eosinophilia [193,194]. These compounds have also exhibited anti-inflammatory effects in other studies, indicating their potential as novel agents in the treatment of eosinophilic asthma. The effectiveness of these compounds in reducing lung eosinophilia and their broader anti-inflammatory actions position them as promising candidates for future therapeutic interventions in eosinophilic asthma.

#### 6.1.9. A Disintegrin and Metalloproteinase 8 (ADAM8)

A disintegrin and metalloproteinase 8 (ADAM8) is expressed on the surface of nearly all types of leukocytes and has been identified as a contributor to the pathophysiology of asthma [195]. The experimental evidence suggests that ADAM8 promotes the intrinsic apoptosis of eosinophils in mice [196], indicating its potential effectiveness in the treatment of eosinophilic asthma. However, further study is required to fully understand the role of ADAM8 and its therapeutic potential in the context of eosinophilic asthma. This line of research holds promise for developing new treatments that specifically target eosinophil-driven inflammation in asthma.

## 7. In Summation

In summary, asthma is a chronic inflammatory disorder of the airways that presents significant challenges, especially in its severe forms, due to its varied phenotypes. The presence of eosinophils in the airways is a major factor contributing to recurrent exacerbations, persistent inflammation, increased severity, and reduced responsiveness to standard anti-inflammatory treatments. The traditional therapeutic methods have often been inadequate in effectively managing eosinophilic asthma, leading to a focus on strategies that target eosinophils.

A new generation of biologic agents has emerged as promising treatments for severe eosinophilic asthma. These include omalizumab, mepolizumab, reslizumab, benralizumab, Lebrikizumab, Tralokinumab, dupilumab, and Tezepelumab. These agents have been effective in reducing asthma episodes and improving patients’ quality of life, though they are not without limitations. Comparative trials evaluating these biologic agents against each other are essential for developing personalized treatment plans for individuals with severe eosinophilic asthma. Such personalized approaches hold the key to optimizing the treatment efficacy and patient outcomes in this challenging subset of asthma.

## 8. Forward-Looking Insights

Looking ahead, targeting a broad spectrum of elements within inflammatory pathways is likely to yield enhanced therapeutic outcomes for all types of asthma, surpassing the efficacy of interventions focused solely on singular cytokines. To accelerate the evaluation of novel treatments and to better understand the relative merits of various biologic agents, the medical community should engage in adaptive platform trials and adopt the principles of pragmatic trial platforms. These initiatives are crucial for developing effective and economically viable therapies for eosinophilic asthma.

In the realm of research, high priority should be placed on identifying biomarkers that can predict the treatment efficacy and patient responses. This includes investigating the immunogenicity risks associated with monoclonal antibodies, determining the optimal duration of biologic treatment, and assessing the safety and effectiveness of these therapies in specific populations, including niche groups and historically underserved communities.

Clinicians on the front lines must remain updated on the evolving guidelines from organizations like GINA (Global Initiative for Asthma). This involves thorough patient assessments, focusing on both phenotypic and endotypic characteristics, and diligently monitoring patient responses to targeted treatments. Such comprehensive and patient-centered approaches are key to advancing the management of eosinophilic asthma and improving patient outcomes.

## Figures and Tables

**Table 1:** Difference between atopic and nonatopic eosinophilic asthma.

Feature	Atopic Eosinophilic Asthma	Nonatopic Eosinophilic Asthma
Trigger	Allergens	Environmental pollutants, glycolipids, and microbes
Age of onset	Childhood	Adulthood (40–50 years)
IgE levels	Elevated, prominent role in B cell activation and production of IgE antibodies	Normal, less evident in IgE-mediated signals
Allergen sensitivity	Present	Absent
Immune cells involved	Th2 cells	ILC2s
Mechanism	IgE-mediated mast cell degranulation	Activation of ILC2 cells by alarmins and leukotrienes
Eosinophil inflammation	Present	Present
Fixed airway obstruction	Late	Early
Associated conditions	Sinusitis, nasal polyposis, NSAID sensitization	None
Specific IgE antibodies in the blood	Positive (at least 0.70 KU/L)	Negative
Skin prick test (SPT)	Positive (papules ≥ 3 mm)	Negative
IFN-γ and IL-8	Contribute to asthma severity	Do not contribute to asthma severity
Cytokines and chemokines	IL-4, IL-13, and IL-5, eotaxins, CCL5/RANTES	IL-4, IL-5, and IL-13, eotaxins, CCL5/RANTES
Corticosteroid response	Good	Poor
Treatment	ICS, LABA, omalizumab	ICS, systemic steroids, other biologics

**Table 2:** Cut-off levels for the absolute number of eosinophils in eosinophilic asthma.

Absolute Count of Eosinophils	Cut-Off Levels
Sputum Eosinophil Count	150/300/400 cells/µL
Blood/Serum Eosinophil Count	2–3% of total cells
BALF	0.5–2% of all analyzed cells
Bronchial Biopsy Samples	5–20 cells/mm^2^
